# Succession of Ephemeral Secondary Forests and Their Limited Role for the Conservation of Floristic Diversity in a Human-Modified Tropical Landscape

**DOI:** 10.1371/journal.pone.0082433

**Published:** 2013-12-11

**Authors:** Michiel van Breugel, Jefferson S. Hall, Dylan Craven, Mario Bailon, Andres Hernandez, Michele Abbene, Paulo van Breugel

**Affiliations:** 1 Center for Tropical Forest Science, Smithsonian Tropical Research Institute, Ciudad de Panamá, República de Panamá; 2 Yale School of Forestry and Environmental Studies, New Haven, Connecticut, United States of America; 3 Forest and Landscape Centre, Faculty of Life Sciences, Copenhagen University, Frederiksberg C, Denmark; University of Massachusetts, United States of America

## Abstract

Both local- and landscape-scale processes drive succession of secondary forests in human-modified tropical landscapes. Nonetheless, until recently successional changes in composition and diversity have been predominantly studied at the patch level. Here, we used a unique dataset with 45 randomly selected sites across a mixed-use tropical landscape in central Panama to study forest succession simultaneously on local and landscape scales and across both life stages (seedling, sapling, juvenile and adult trees) and life forms (shrubs, trees, lianas, and palms). To understand the potential of these secondary forests to conserve tree species diversity, we also evaluated the diversity of species that can persist as viable metapopulations in a dynamic patchwork of short-lived successional forests, using different assumptions about the average relative size at reproductive maturity. We found a deterministic shift in the diversity and composition of the local plant communities as well as the metacommunity, driven by variation in the rate at which species recruited into and disappeared from the secondary forests across the landscape. Our results indicate that dispersal limitation and the successional niche operate simultaneously and shape successional dynamics of the metacommunity of these early secondary forests. A high diversity of plant species across the metacommunity of early secondary forests shows a potential for restoration of diverse forests through natural succession, when trees and fragments of older forests are maintained in the agricultural matrix and land is abandoned or set aside for a long period of time. On the other hand, during the first 32 years the number of species with mature-sized individuals was a relatively small and strongly biased sub-sample of the total species pool. This implies that ephemeral secondary forests have a limited role in the long-term conservation of tree species diversity in human-modified tropical landscapes.

## Introduction

Today’s tropical landscapes are mostly dynamic mosaics of mature forest remnants, crop land, pastures, and regrowing secondary forests of different ages. In many tropical regions, young secondary forests now cover larger areas than mature forests [1]–[3] and increasingly define the prospects of long-term conservation of ecosystem services and biodiversity [4], [5].

While changing socio-economic circumstances have led to long-term forest recovery over considerable areas in some regions [3], [6]–[8], secondary forests more often are an integral component of agricultural land-use systems. In these systems fallows form a dynamic patchwork of early successional plant communities across the landscape and rarely surpass a few years or decades in age [9]–[13]. Developing effective conservation plans for multi-functional landscapes requires an understanding of the value of transient secondary forests in maintaining plant diversity [14], [15]. It is therefore imperative that we examine successional patterns in the composition and diversity of early secondary forest communities as well as those of the metacommunity [16] of these secondary forests.

Until now, tropical secondary forest succession has been studied predominantly at patch level. The classical successional niche-based model of forest succession is that of a deterministic change in the species composition of a local community, driven by changes in environmental conditions during stand development [17]–[19]. In moist tropical forests, light availability and a tradeoff between fast growth under high light conditions and the ability to establish and survive in low light conditions are typically considered drivers of early successional species turnover [20]. However, models based on local niche-based processes do not predict landscape scale patterns of diversity and composition nor how these change during succession. Metacommunity models, in contrast, provide a framework in which dispersal limitation and local environmental conditions interact, through trade-offs between fecundity and colonization and tolerance or competitive abilities, to determine local- and landscape-scale variation in diversity and composition [16], [21]–[23].

In this study, we examine the extent to which both dispersal limitation and local niche-based processes drive deterministic succession trajectories of secondary forests on local and landscape scales. We used a novel sampling design with a large number of randomly selected 0.2 ha sample sites across a 15 km^2^ study area that represent a highly replicated sample of the variation in age, stand structure, and composition of secondary forests across this landscape.

We first evaluated – simultaneously across plant life stages and life forms – if successional changes in diversity and composition of local communities and of the metacommunity of secondary forests were predictable. Changes in community structure may emerge from species turnover and internal community re-ordering that result in shifts in relative abundances [24]. To capture both processes, we quantified community structure using a range of diversity and similarity indices that differ in the weight given to species number, abundance, and identity. Then, we compared the composition of seedlings and larger stems to test if recruitment of early dominants was limited in later successional stages. Finally, we analyzed successional changes in landscape-scale patterns of frequency and relative dominance to evaluate how variation in the patterns and rates of species dispersal across the metacommunity leads to changes in diversity and composition.

The question of whether secondary forests can recover the diversity and composition of mature forests has recently generated an intense debate [4]–[6], [14], [15], [24]–[26]. However, in agricultural landscapes a more pertinent question with respect to biodiversity conservation is to what extent a metacommunity of transient successional plant communities constitutes a source rather than a sink for tree species [27]. In other words, what proportion of the tree species reaches reproductive maturity within a fallow period and what are the implications for the prospects of long-term conservation of tree species in human-modified tropical landscapes? This is particularly relevant as increased demand for land and natural resources lead to increased pressure on mature forest, shorter fallow periods, and agricultural intensification. To explore these questions, we evaluated the diversity of species that can persist as viable metapopulations in different successional age classes, using different assumptions about the average relative size at reproductive maturity.

## Methods

### Statement of Ethics

Permission for collection of plant and soil samples was provided by the National Environmental Authority of Panama (ANAM). The Agua Salud land is managed by the Smithsonian Tropical Research Institute and outside permission to conduct the study on this site was thus not required.

### Data availability

Data from this study are archived in the Smithsonian Institution Forest Global Earth Observatory (ForestGEO) database and available at http://ctfs.arnarb.harvard.edu/Public/plotdataaccess/.

### Study Site and vegetation inventories

**Figure 1:**
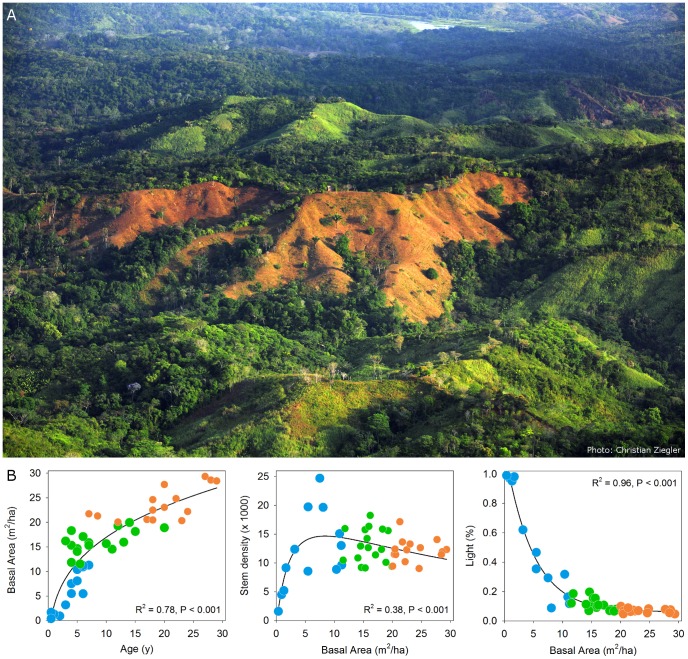
The Agua Salud landscape and its secondary forests. A) An aerial overview of part of the Agua Salud landscape, with a mosaic dominated by secondary forests of different ages and pastures. Note the isolated trees and live fences in the pastures and the strips of forest along the streams. Photo printed under a CC BY license with permission from Christian Ziegler. B) Predictable changes in stand and canopy structure of secondary forests during succession, but with substantial variation among similar aged sites. Light  =  the average light availability in the understory as a percentage of above-canopy values and was calculated from 11 hemispherical photographs per plot. Blue, green and orange colors indicate SFD plots with stand basal areas of 0-11, 11-20, 20-30, and >30 m^2^ ha^−1^, respectively. See Fig. S1 for the distribution of the SFD plots across the landscape.

The 15 km^2^ “Agua Salud“ study area is located adjacent to Soberania National Park in the central part of the Panama Canal Watershed (9°13' N, 79°47'W, 330 masl, Fig. S1). Annual rainfall is 2700 mm, with a dry season from mid-December through early May [28]. Topography is undulating with short, steep slopes intersected by a high density of narrow streams. Soils are well drained, nutrient poor Oxisols that exhibit little variation in texture (silty clays to clays) (Table 1) [29]–[31]. The flora and vegetation of the region are described by Croat [32] and Leigh [33]. Since the 1950s, land use in the study area has been dominated by extensive cattle ranching and small-scale shifting cultivation. Presently, the landscape consists of a mosaic of cattle pastures and cultivated fields, fallows, secondary forests, plantations and fragments of older secondary forest [34](Fig. 1a). Within the study area, the Smithsonian Tropical Research Institute (STRI) manages 664 ha of land (henceforth "Agua Salud "), of which ∼530 ha is covered by fallow vegetation and forests of various ages (Fig. S1).

**Table 1:** Mean Soil Nutrient Concentrations across the Agua Salud study area.

Nutrient	Unit	Mean	95% C.I.
P	ppm	1.20	1.19–1.20
K	ppm	94.3	93.3–94.3
Ca	ppm	929	918–941
Mg	ppm	371	367–377
Cu	ppm	3.01	2.99–3.04
Mn	ppm	204	202–207
Zn	ppm	2.90	2.86–2.94
Fe	ppm	99.0	98.0–99.5
N	%	0.27	0.27–0.27

± 95% confidence intervals calculated from one bulked samples per SFD plot (n  =  90). Per plot five samples were taken at 0–15 cm depth. Plant available phosphorus and base cations were extracted using the Mehlich III method [95]. Notes: Mean

We chose 52 sites at random within the forested area of Agua Salud (Fig. S1), which thus represented an unbiased sample of existing landscape-wide variation in stand age, - structure and - composition. A site was defined as a single slope (segment) within a former agricultural field or cattle pasture. To account for variation in the structure of the plant community along the slope, we established a 20×50 m plot on the upper portion and another plot on the lower portion of the same slope. Plot orientation was perpendicular to the main slope direction. After plot establishment, information on the time since abandonment of the sites was obtained from interviews with former land owners and local residents. The age of the five oldest sites was estimated to range between 50 and 80 years, but no information was available to confirm this estimate. Furthermore, no reliable information was available on forest use since abandonment. We therefore decided to exclude these sites from analyses. In four sites we found that the age of the vegetation on the upper and corresponding lower slope differed markedly, as they likely originated in different agricultural fields or cattle pastures. These could therefore not be used as single sites and were excluded from site-level analyses. We thus used a total of 45 secondary forest dynamic (SFD) sites and 98 SFD plots for analysis. The age of sites included in the analyses varied from 2 to 32 years, with a fairly even distribution along this range (Fig. 1b). We used sites, pooling pairs of plots, for most analyses but used plots for analysis of the landscape-scale frequencies of species (see below).

In each plot, all trees, shrubs, and palms with a diameter at breast height (DBH) ≥ 5 cm and woody climbing plants with a diameter of ≥ 1 cm were tagged, measured, and identified to species. In one half of each plot, all woody, non-climbing plants with 1–5 cm DBH were treated similarly (Fig. S2). Stem diameters of lianas were measured following Gerwing et al. [35] and Schnitzer et al. [36] and diameter of all other plants was measured at 1.3 m height. Plants 20–80 cm high, including all tree, shrub, palm, and liana species and *Carludovica palmata*, the only species of the family Cyclanthaceae, were sampled in 20 1×1 m sub-plots (Fig. S2). For convenience, we will refer to these plants as ‘seedlings’, to woody climbing plants as ‘lianas’, and to all woody non-climbing plants with 1–5 cm DBH and >5 cm DBH as ‘saplings’ and ’trees’, respectively. Across all plots, 7,262 seedlings, 26,490 saplings, 9,906 trees and 10,643 lianas were found. Over 98% of individuals were identified to species. Voucher specimens that were collected were stored at the herbarium of the Smithsonian Tropical Research Institute (Index Herbarium code: SCZ). A list of all species with data on life form (trees, lianas, palm or other) and their total abundances across all 45 SFD sites is given in Table S1.

We used a chronosequence approach to evaluate landscape scale changes in the composition and diversity of local communities and of the metacommunity of these local communities [16]. The latter was done by grouping the 45 SFD sites in age classes and site basal area (SBA) classes, with a similar number of sites per class. SBA classes were: SBA-1, 1–11 m^2^ha^−1^ with 13 SFD sites; SBA-2, 11–20 m^2^ha^−1^ with 17 sites, and SBA-3, 20–30 m^2^ha^−1^ with 15 sites (Fig. 1b). Age classes were 2–7 y, 8–17 y, and 18–32 y, and all classes had 15 sites.

Although this study involves a highly replicated chronosequence, it is still important to keep in mind that we substitute space for time, i.e. infer temporal trends from static data. Potential limitations and caveats to this approach have been extensively discussed elsewhere and need to be taken into account in the interpretation of our results [37]–[40].

### Composition

To assess variation in species composition of the four plant groups, we employed non-metric multidimensional scaling (NMDS) and calculated the positions of all sites along the ordination axes. NMDS ordinations were based on similarity matrices generated from both the Jaccard abundance-based (or Ružička) index and the presence/absence version of the same index [41], [42]. Calculations were done using the function ‘metaMDS’ of the R package ‘vegan’ [41], with several random starting configurations to find stable convergent solutions. The variance of points along the first axis was maximized by standardizing the scaling by a principle components rotation. We chose 3 or 4 dimensions based on visual inspection of scree plots of stress values. Stress values were below 0.10 in most cases, except for seedlings where 4 dimensions resulted in a stress value of 0.13.

To understand the extent to which the relative positions of the sites along the ordination axes were determined by the distribution of the most frequent species, separate NMDS were performed for species that occurred in more than 10%, 25%, and 50% of the sites. For each frequency threshold, site scores were correlated with site scores calculated from the full data set.

To assess if dissimilarities in species composition was related to spatial distance, we calculated mantel statistics separately for each of the twelve plant group × age classes and the twelve plant group × SBA classes. The species dissimilarity matrices were calculated with the ‘vegdist’ function of the vegan package [41], using the Jaccard abundance index. Mantel statistics were calculated using the function ‘mantel’ and ‘mantel.correlog’ of the R package ‘vegan’ based on Pearson's product-moment correlation. Significance was evaluated by permuting rows and columns of the species dissimilarity matrix 999 times [41].

We repeated all analyses using other similarity indices. The results were similar and therefore only results obtained with the Jaccard abundance and presence/absence based indices are reported.

### Diversity

We used Hill numbers for measuring different aspects of diversity that differ in their sensitivity to the species number and evenness components of diversity [43]–[45]. When there are S species and relative abundance of the i^th^ species is p_i_, the Hill number of order *q* is [45], [46]:



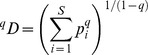



The Hill number is undefined for 1, but:



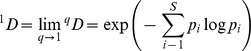



The scale parameter q defines the sensitivity of the diversity measure to the common and rare species by modifying how the mean of the species proportional abundances is calculated [45], [46]. We calculated ^0^D  =  S, which is species density; ^1^D which is the exponential of Shannon entropy and weights species proportionally to their abundances [43]; and ^2^D which is the inverse Simpson concentration. The latter calculates the arithmetic mean of species abundances and thus favors abundant species disproportionally. Hill numbers give the “effective number of species” or “true diversity” of a community [47], [48]: When ^q^D  =  *n*, then the ^q^D of the observed community is the same as that of a hypothetical community of *n* equally-common species [45]–[48]. Hill numbers were calculated using the function ‘renyi’ of the R package ‘vegan’ [41].

All diversity measures were calculated for individual SFD sites (α-diversity) and for the pooled plots in each SBA and age class (γ-diversity). Henceforth, we will use the subscripts ‘α’ and ‘γ’ in combination with the indices ^q^D when referring to site-level or landscape-level diversity. To arrive at comparable indices for SBA classes with a different number of sites, we used resampling procedures with multiple iterations where random samples of 12 SFD sites were selected 1000 times. For each of these samples, ^q^D_γ_ was calculated and means and confidence limits were determined, the latter by calculating the 0.975 and 0.025 quantiles from the resampling distribution [49]. The mean number of species per 12 SFD sites (^0^D_γ_) was obtained from sample-based species accumulation curves using the function ‘accumresult’ of the R package ’vegan’.

To enable a direct meaningful comparison between the diversity of the different plant groups, we calculated rarefied species richness (^rf^S_α_ and ^rf^S_γ_) for a given number *n* of individual plants, where *n* was the lowest number of individuals among all plant group × plot combinations (^rf^S_α_) or plant group × SBA class combinations (^rf^S_γ_) [50]. Rarefaction was performed using the function ‘rarefy’ of the R package ’vegan’ [41].

### Successional changes in community structure

We tested for deterministic change in the community structure of secondary forests by relating ^q^D_α_, ^rf^D_α_ and the scores of the SFD sites on the NMDS axes (composition) to age and stand basal area using linear and non-linear regression. Stand basal area was included as a predictor variable as it integrates many aspects of plant response to changing environmental conditions over time [51], [52]. In the present study, we found that the use of site basal area consistently resulted in statistically stronger patterns and hence we focus on this explanatory variable in the main text.

We examined successional changes in γ diversity by comparing the three SBA classes [53]. To compare variation in composition within versus between SBA classes, we used a non-parametric MANOVA, an analysis of variance based on distance matrices [54], using the adonis function in ‘vegan’ [41].

We used the Chao-Jaccard abundance-based estimator[55] to compare similarities in canopy tree species composition of the seedling assemblages in SBA class 1, 2 and 3 with that of the assemblages of larger stems in SBA-1. Canopy tree species were defined as species that potentially can grow to ≥ 5 cm diameter (see below for an explanation on how species were classified according to maximum size). Given the small sample sizes of the seedlings compared to the larger stems, the Chao-Jaccard estimator is considered better suited than other commonly used indices as it accounts for the effect of unseen, shared species and therefore is less biased by sample size [55]. Using the Morisita-Horn index [56], [57] gave similar results and,thus, is not reported here. Pairwise similarities were estimated using the function ‘vegdist’ of the R package ‘vegan’ [41] and a mean and confidence limits were calculated for each of the three comparisons.

### Landscape-level frequencies of occurrence

To assess successional patterns in species abundance across the landscape, for each species of all plant life forms we calculated the frequency of occurrence in SFD plots with at least one individual ≥ 1 cm diameter (F_1_) as well as with ≥ 5% of total number of individuals ≥ 1 cm diameter (_F5%_). To evaluate how successional changes in landscape-scale frequencies varied among species, we calculated F_1_ and F_5%_ in each SBA class and plotted the values of the different SBA classes against each other (e.g., F_5%_ (SBA-1) vs. F_5%_ (SBA-2)).

### Diversity above different relative plant size thresholds

To assess the potential of secondary forest to conserve reproductive metapopulations of tree and shrub species, we used different relative size thresholds as proxies for reproductive size and counted the number of tree and shrub species across all 45 SFD sites above these relative size thresholds. To this end, we used our own data and the Smithsonian Forest Global Earth Observatory database (ForestGEO, http://ctfs.arnarb.harvard.edu/webatlas/datasets/bci/) with over 400,000 trees from census plots and inventories across central Panama [58] to classify all species in the following maximum DBH (DBH_max_) classes: 1–5; 5–10; 10–20; 20–40; 40–80; >80 cm DBH. As a control, three expert field botanists with decades of experience in Panama classified species in the indicated size classes. We then used the mid-value of each class as a conservative estimate of DBH_max_ of each species and determined the relative size of all individual trees. We compared the total number of species with the number of species above the relative size threshold of 10% and 30% (RST_10%_ and RST_30%_) and we compared this ratio among the six DBH_max_ classes and the three age classes. RST_30%_ was chosen based on the results of a study by Wright et al. [59] in a nearby mature forest in which they estimated relative size at onset of reproductive maturity (DBHthr) for 16 tree species. RST_10%_ was chosen to assess a more optimistic scenario.

## Results

### Successional patterns in species composition

**Figure 2:**
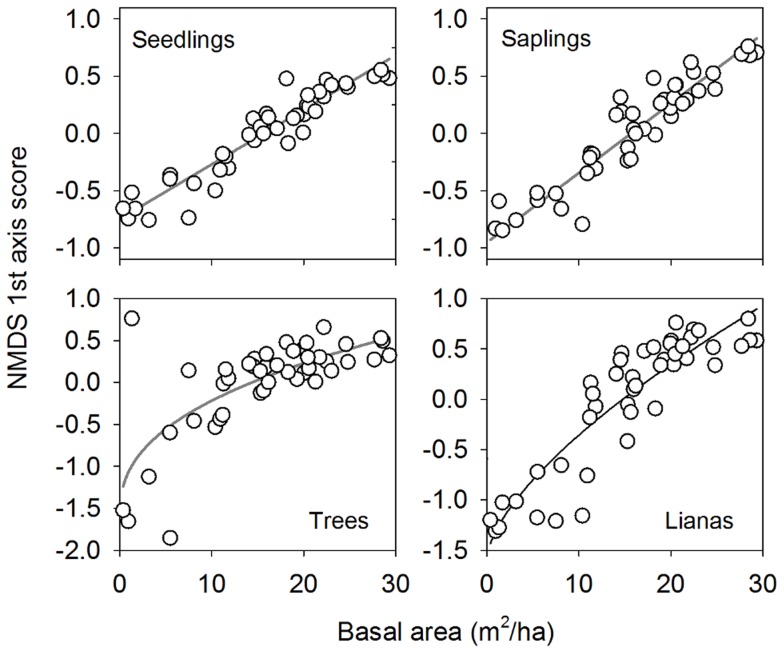
Compositional change with successional stand development. Variation in species composition was assessed for four different groups of woody plants by calculating the relative positions of sites along the axes of a non-metric multidimensional scaling (NMDS) ordination, based on the Jaccard abundance-based dissimilarity index. See methods for definition of plant groups and sample areas. Equation type and regression statistics are provided in Table 2.

Over 80% of the variance along the first axis of the NMDS ordination was explained by stand basal area and 60–75% by forest age for seedlings, saplings and lianas (Table 2, Fig. 2 and Fig. S3). For trees, this was substantially lower (47% and 31%, respectively), reflecting large variation among the youngest plots. In these plots, very few individuals exceeded 5 cm DBH limit and consequently played a disproportionately large role in determining plot scores.

**Table 2:** Regression models and coefficients of determination for the relationships between indices of community structure (response variables) and age since abandonment, stand basal area (SBA), or light (predictor variables).

Diversity	Plant group	Age		SBA		Light	
		M	R^2^	M	R^2^	M	R^2^
^0^ *D*_α_	Seedlings	P	0.27	P	0.44	E	0.31
	Saplings	P	0.55	P	0.72		
	Trees	P	0.71	P	0.85		
	Lianas	L	0.79	L	0.79		
^1^ *D*_α_	Seedlings	P	0.23	L	0.39	E	0.43
	Saplings	P	0.43	L	0.61		
	Trees	L	0.57	P	0.65		
	Lianas	P	0.30	L	0.39		
^2^ *D*_α_	Seedlings	L	0.14	L	0.25	-	*ns*
	Saplings	L	0.31	L	0.49		
	Trees	L	0.47	P	0.50		
	Lianas	-	*ns*	-	*ns*		
Composition (1)	Seedlings	P	0.71	L	0.89	E	0.80
	Saplings	P	0.75	L	0.88		
	Trees	P	0.31	P	0.47		
	Lianas	P	0.60	P	0.81		
Composition ^(2)^	Seedlings	P	0.71	L	0.89	E	0.80
	Saplings	P	0.76	P	0.76		
	Trees	P	0.38	P	0.57		
	Lianas	P	0.63	P	0.63		

^(2)^ calculated with presence/absence data; M  =  Model. P  =  power model; L  =  Linear model; E  =  Exponential model. *ns*  =  non-significant relationship (p>0.05), in all other cases significance is p ≤ 0.01.^(1)^ Calculated with abundance weighted data;

Patterns and coefficients of determination were very similar when dissimilarity among sites was computed with presence/absence data (Table 2, Fig. S3). This suggests that much of the deterministic change in community structure is driven by species turnover rather than consistent successional changes in the relative abundances of species. The first-axis site scores of NMDS based on species that occurred in at least 10%, 25%,or 50% of the sites were strongly correlated to the site scores calculated from the full data set (R^2^ >0.90). Thus, the coordination among sites scores along the first site axis was principally driven by a small group of very frequent species that occurred in at least half of the sites.

It is nevertheless important to note that a large proportion of the compositional variation was unrelated to either age or stand structure, with no significant relationship between site scores on the other ordination axes and these variables. In a non-parametric MANOVA, SBA classes explained a small amount of compositional variation in the whole metacommunity (Seedlings: F  =  7.58, R^2^  =  0.15; Saplings: F  =  8.94; R^2^  =  0.17; Trees: F  =  5.70, R^2^  =  0.12; Lianas: F  =  8.25, R^2^  =  0.16; df  =  1 and p < 0.01 in all cases). Spatial distance explained a modest proportion of the variation in compositional dissimilarities among plots, but only in the earliest successional stage. In the 2–7 y age class, overall mantel correlations were 0.17, 0.27, 0.14 and 0.33 for seedlings, saplings, trees and lianas, respectively. In SBA class 1, values were similar (Fig. S4). Spatial correlations were only significant over short distances (< 300 m, fig. S4).

**Figure 3:**
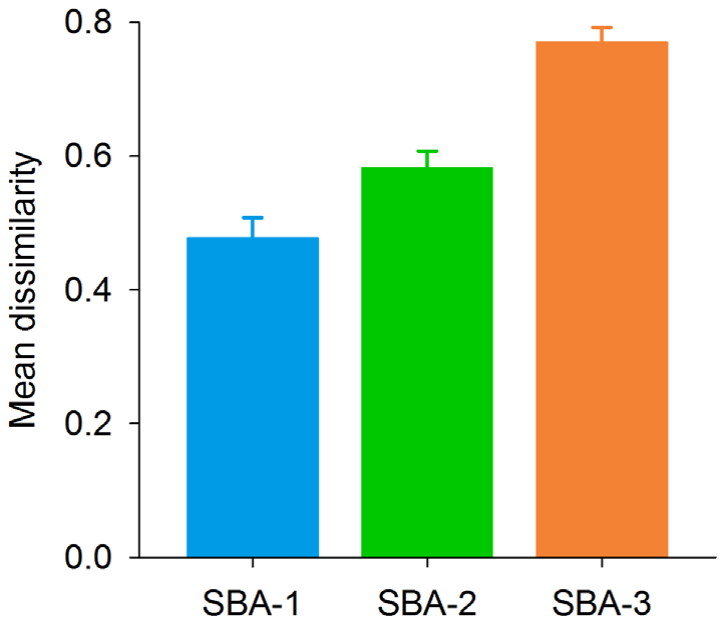
Increasing dissimilarity between seedling and the initial assemblages of trees. Mean of pair-wise dissimilarities between the seedling assemblages of SBA classes 1, 2 and 3 with the assemblages of plants ≥ 1 cm DBH of SBA class 1, using the Chao Jaccard Abundance Estimator. Error bars indicate ± 95% confidence limits. Calculations included only species that can potentially grow to a diameter of at least 5 cm.

Similarities between the canopy tree composition of the seedling communities and the communities of larger stems were fairly high in SBA class 1, with an average pair-wise similarity of 0.52 (Chao-Jaccard abundance estimator) and decreased in subsequent SBA classes to an average of 0.42 and 0.22 in SBA class 1 and 2, respectively. Seedling composition in SBA class 2 and 3 increasingly diverged from the composition of larger stems in SBA-1, suggesting an ongoing landscape-scale successional turnover of canopy tree species (Fig. 3).

### Successional patterns in diversity

**Figure 4:**
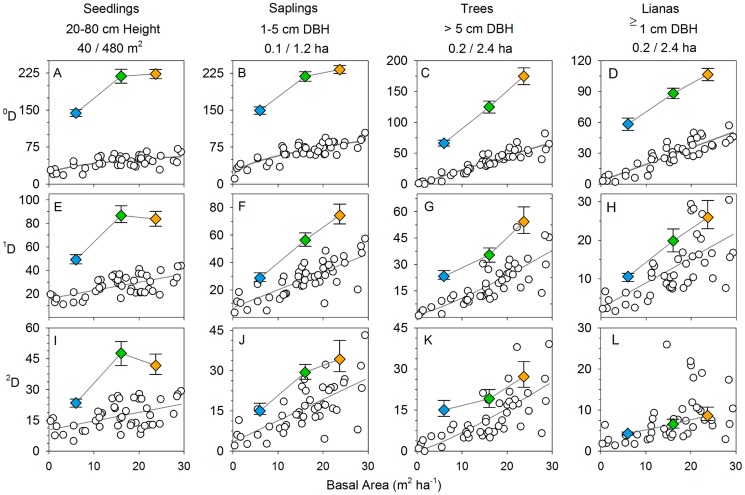
Changes in diversity with successional stand development on local and landscape scale. Indices of species diversity were calculated for four different groups of woody plants, and for individual sites (white dots and regression lines) and the metacommunity (diamonds and triangles). A-D) ^0^ *D*  =  species density. E-H) ^1^ *D*  =  the exponential of Shannon entropy. I-L), ^2^ *D*  =  the inverse Simpson concentration. For all three diversity measures, units are in number of species. Metacommunity diversity was calculated for the pooled data of randomized samples of 12 SFD sites. Colors indicate SBA classes as in figure 1 and error bars give the 95% confidence limits. The lines connecting the symbols are for illustrative purposes only. Sample area per plot and per SBA class is indicated above the graphs. Equation type and regression statistics are given in Table 2.

The proportion of explained variance in the diversity of secondary forests attributable to age or stand basal area varied among plant groups and also depended on which diversity indices were used (Table 2, Fig. 4). Species densities (^0^D_α_) of saplings, trees and lianas increased rapidly during the first 30 years of succession and had high coefficients of determination for both age and SBA (Table 2). Species densities of the seedling assemblages varied less predictable with successional stand development (Fig. 4a-d). Diversity changed less predictably with age and SBA when diversity measures stronger emphasized the more abundant species (Fig. 4e-l, Fig. S3). Likewise, rarefied species richness, which accounts for differences in stem densities, yielded similar successional patterns of increasing diversity, but with relatively low coefficients of determination (Fig. S5). Overall, these results indicate that successional change in α-diversity was characterized predominantly by a continuous recruitment of new species rather than a predictable change in the number and relative abundance of locally dominant species.

**Figure 5:**
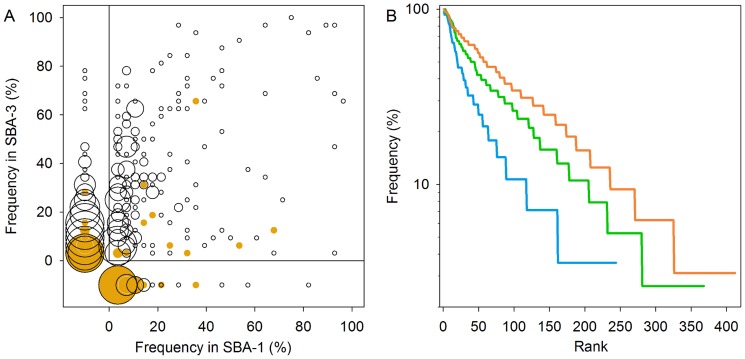
Frequency of occurrence in stand basal area (SBA) classes. Frequency is calculated as the percentage of the plots in which a species occurred with individuals ≥ 1 cm diameter. Species include trees, shrub, palm and lianas. A) A scatter plot of frequencies in SBA class 1 and 3. Open circles give frequencies with ≥ 1 individual (F_1_) and yellow dots give frequencies with ≥ 5% of total number of individuals (F_5%_). The size of the circle indicates the number of species with the indicated frequencies (indicated range: from 1 to ≥ 10 species, true range: 1–75 species). Frequencies of species that occurred in one of the SBA classes, but not the other, are plotted to the left or below the null axes. B) Rank-frequency curves, colors indicate SBA-1 (blue), SBA-2 (green) and SBA-3 (orange).

Successional patterns in γ-diversity were qualitatively similar to patterns in α-diversity. The average number of species per 12 SFD plots (^0^D_γ_) ranged between 58 and 232 species and increased continuously during succession in the cases of saplings, trees, and lianas. Seedling diversity only increased during the earliest phase of succession (Fig. 4, S5e). The differences between α and γ diversity decreased when more weight was given to the very abundant species. This reflects a more uneven distribution of relative abundance among species at the scale of the metacommunity, with a large number of infrequent species and relatively few species that are frequently abundant across the local communities (see next section, Fig. 5).

### Frequencies of occurrence across the landscape

During the first 32 years of succession relatively few species disappeared, while a much higher number of new species recruited into the metacommunity. Whereas 12% and 16% of the species that were found in SBA-1 did not occur in SBA classes 2 and 3, respectively, 41% of the species in SBA-2 and 50% of the species in SBA-3 were not found in SBA-1.

Shifts in frequencies between SBA classes varied strongly among species (Fig. 5a). For example, of the 20 species that had an F_1_ ≥ 50% in SBA class 1, eight species were less than half as frequent in SBA class 3, most of which were short-lived and low-statured pioneers. At the same time, the majority of the species in SBA1 (67%) increased their frequency, i.e. F_1_(SBA3) > F_1_(SBA1). One consequence was that the number of species with F_1_ ≥ 50% increased to 62 in SBA class 3 and the slope of the rank-frequency curve decreased during succession (Fig. 5b). In other words, a large proportion of the trees that recruited early in succession in some of the sites of the metacommunity continued recruiting into new sites, but with strong interspecific variation in recruitment rates.

As noted above, local abundances of species had no or very little effect on the patterns of deterministic species change during succession. This was because very few species were frequently locally abundant: Only 74 species (out of 481 tree, shrub, liana and palm species in 98 SFD plots, Table S1) had relative abundances of ≥ 5% in at least one plot. Moreover, most of these species were abundant in one plot (29 species) or in one SBA-class (39 species) only. Only a handful of species occurred with more than 5% of the stems in at least one third of SFD plots (5, 3 and 2 species in SBA class 1-3, respectively) and only two species, *Vismia baccifera* and *Davilla nitida*, reached these thresholds in at least two SBA classes (Table S2).

Our landscape perspective, in which space substitutes for time, emphasizes species differences in frequencies across the metacommunity. It is important to note that, when secondary forests are followed over time, successional changes in individual sites may well be mostly due to changes in relative abundance over time, at least over the time span of our chronosequence [60].

### Diversity above relative size thresholds and under different land-use scenarios

**Figure 6:**
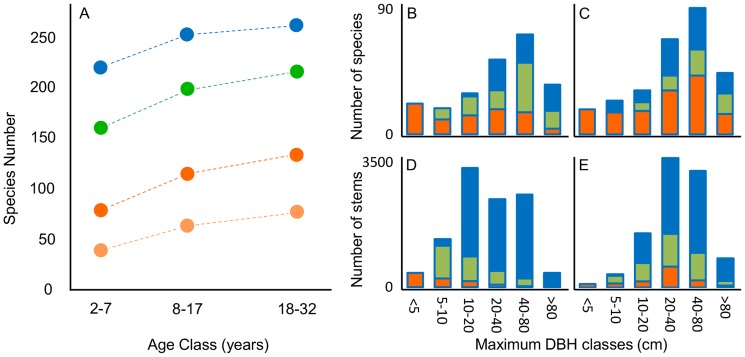
Diversity in relationship with relative size and forest age. A) Number of tree and shrub species per age class (lianas and palms not included!), when all individuals are taken into account (blue dots), when only individuals above the relative size threshold (RST) of 10% (green dots) or of 30% (darker orange dots) are counted and when only species are counted with individuals ≥ RST_30%_ in more than one plot (lighter orange dots). Per age class, species were counted for the pooled data of 15 SFD plots (3 ha). Dotted lines are for illustrative purposes. B-C) Number of species per maximum-size class (blue) and the subsamples of species with individuals above relative size thresholds of 10% (green) and 30% (orange). D-E) Number of stems per maximum size class. Graphs show data for the 2–7 y age class (B, D) and the 18–32 y age class (C, E).

The γ-diversity of tree and shrub species in the secondary forests in our study area was high, with 324 species in 45 SFD plots (liana and palm species not included). This number represents 55% of all tree and shrub species encountered in the Agua Salud area in this study as well as extensive inventories of older forest patches, stream side vegetation, and pastures (van Breugel & Hall, unpublished data). We used different relative size thresholds to estimate the proportion of these species with mature-sized individuals in the different age classes (henceforth ‘effective γ-diversity’). When assuming a community average relative size at reproductive maturity of 30% (RST_30%_), the effective γ-diversity was 64% lower than the total γ-diversity in the youngest age class (2–7 y, 220 species), and 49% lower in the oldest age class (18–34 y, 268 species)(Fig. 6a). When assuming a much lower relative size threshold of 10%, these values were 27% and 18%, respectively. The large differences between these estimates emphasize the importance of obtaining data on reproductive size in relationship to size and environmental conditions.

Land-use dynamics, and more specifically fallow length, may not only affect the number of species that can complete their life cycle in the metacommunity of secondary forests, but also alter the functional composition of these forests. Here, we looked at the distribution of DBH_max_ among tree species. Even in the youngest age class, the largest group of species and stems in our metacommunity were mid-canopy and canopy species (DBH_max_  =  20–40 cm, 40–80 cm and >80 cm)(Fig. 6b-e). Across these three DBH_max_ classes, on average a mere 22% of all species had individuals above RST_30%_ in the 2–7 y age class (Fig. 6b) and 42% of all species in the 18–34 y age class (Fig. 6c). These averages were 67% and 73%, respectively, for the three lower DBH_max_ classes, which illustrates that the functional composition of the subset of reproductive species is potentially very different from the functional composition of the complete set of observed species in early successional forests.

In a dynamic agricultural matrix interspersed with short-lived secondary forests, landscape-scale abundance will be an important factor in the probability of arriving at suitable sites. Overall, the actual number of individual trees that surpassed RST_30%_ was very low in all age classes and in all but the smallest DBH_max_ classes (Fig. 6d,e). Removal of the very-infrequent species (occurring in one site only) from species counts illustrates that our estimate of the effective γ-diversity may still be strongly inflated when the rareness of species is not taken into account (lighter orange dots, Fig. 6a).

## Discussion

Tropical secondary forest succession in human-modified landscapes has been widely studied empirically at the scale of individual plots, but seldom at the scale of the metacommunity. Our study demonstrates that both scales of observation are essential for understanding processes that control plant community assembly during succession. Patterns of diversity and composition across our landscape were characterized by a deterministic successional shift in the composition of plant communities across the landscape. Detailed analyses of these patterns strongly support the idea that metacommunity dynamics of early secondary forests are simultaneously shaped by dispersal limitation and the successional niche.

### Predictable changes in composition and diversity during tropical forest succession

To our knowledge, this is the first study to assess changes in the diversity and composition of a metacommunity of secondary forests during succession [61], [62], [63]. Our data show that an important part of the compositional variation across the metacommunity of secondary forests in the Agua Salud area is structured along a successional gradient, with early successional sites that are compositionally more similar to each other than to later successional sites and vice versa. Seedlings, saplings, trees, and lianas all followed similar patterns (Fig. 2).

An important driver of this directional change in species composition was the continuous recruitment of new species in local communities and across the metacommunity during the first 30 years of succession (Fig. 5), This resulted in a few clear successional trends in diversity: First, there was a rapid and predictable increase in local and landscape-scale species densities (^0^D, Fig. 4a-d). Diversity measures that weigh abundant species proportionally (^1^D) or disproportionally (2D) varied less predictably during succession (Fig. 4e-l), indicating a much less consistent change in local dominance. Second, on the scale of the metacommunity, the number of frequent to very-frequent species increased significantly during succession (Fig. 5). As a consequence, the proportion of rare species (frequency ≤ 2 sites) in the total species pool decreased from 52% to 37%, despite an increase in their absolute number from 127 early in succession to 161 later in succession.

In the following two sections, we will examine in more detail how both local niche-based processes and species differences in dispersal limitation may have shaped these observed patterns of diversity and composition across the metacommunity.

### Local processes and the successional niche

The ‘successional niche’ hypothesis predicts the sorting of species with similar niche requirements along an axis of successional change in the abiotic and biotic environment [17], [18]. A crucial prediction of this hypothesis is that species that dominate youngest sites regenerate poorly in older sites where understory light availability is strongly reduced (Fig. 1b) and are gradually replaced by more shade tolerant species. Indeed, we found strong dissimilarity between the seedlings of later successional sites and trees of the earlier successional sites, indicating poor establishment of most of the species that were very frequent in the earliest stage of succession later in succession (Fig. 3). These and similar findings elsewhere provide consistent empirical evidence for the importance of the successional regeneration niche during humid tropical forest succession [64]–[66].

We also expect that the initially dominant species disappear from a metacommunity as successional stand develops and competition increases. Indeed, the frequency of some of the initially very-frequent species strongly declined, suggesting a gradual disappearance from the metacommunity. This group consisted of small and short-lived pioneer species, such as *Vernonanthura patens*, *Piper aduncum*, and *Conostegia speciosa*. These species can proliferate in recently abandoned sites because high fecundity and long dispersal allow them to colonize a site before stronger competitors arrive. Moreover, rapid growth under high light allows them to temporarily outgrow later successional species that arrive at the same time. Other species that occurred in over 50% of the youngest sites remained frequent in later successional stages, such as *Xylopia frutescens*, *Terminalia amazonia*, and various species in the *Vismia* and *Miconia* genera. The disappearance of some and persistence of other initially frequent species indicates a range in the capacity to tolerate increasing competition during succession [67], as well as differences in life span, among the early dominants.

### Landscape scale recruitment patterns and dispersal limitation

The design of our study system allowed us to quantify frequency patterns across a metacommunity of tropical secondary forests and how these patterns changed during succession. From these patterns we infer how successional changes in composition and diversity were driven by interspecific variation in the rate at which species recruited into - and disappeared from secondary forests across the landscape.

A low number of species recruited quickly in most sites early in succession, while the rest of the species recruited at slower and more variable rates across the metacommunity (Fig. 5). A crucial detail is that the majority of species were not restricted to recruiting during specific stages of succession, but rather gradually increased their frequency over the course of succession. This pattern and the broadly similar soil conditions across our sites (Table 1)[31], [68] suggests that interspecific variation in dispersal limitation – the failure of species to arrive rapidly in all sites across the meta-community that are suitable for their establishment, growth and survival [69] – is a major determinant of compositional change during succession.

Our results beg the question: how would dispersal limitation drive predictable patterns in successional species change? The accumulated probability of arrival at different sites will increase over time for all species, but at very different rates for different species. The differential ability of a species to disperse across the metacommunity depends on a suite of species traits and life history characteristics, including fecundity, seed size, dispersal mode, and dispersal vectors [70], [71]. For the large group of animal-dispersed species, dispersal to a site will be increasingly facilitated during succession as the forest develops and becomes a more attractive habitat for their dispersers [70], [72]–[74].

High fecundity and a good dispersal ability, as well as fast growth under favorable (light) conditions, are hypothesized to trade off against survival under low-light conditions, and both trade-offs can thus be expected to operate in parallel [21]. While much emphasis has been given to the role of the growth-mortality trade-off in driving deterministic tropical forest succession, few studies have tried to empirically link compositional change during succession to a tradeoff between shade tolerance and seed production and dispersal [23]. In studies that focus on local processes, dispersal and colonization are often considered to be stochastic processes [20]. However, our data support a metacommunity model in which species-specific differences in dispersal limitation are a strong contributing force to a deterministic successional shift in the composition of the local communities and the whole metacommunity.

One important caveat is that we cannot exclude the possibility that the observed differences in species frequencies reflect differences in initial composition rather than a shift in composition. For example, recruitment patterns may have changed over the period covered by the chronosequence due to changes in land-use dynamics. Ultimately, a combinations of highly replicated landscape-scale sampling schemes with permanent study plots will provide more details on the processes that drive compositional change and species’ changes in frequency/abundance on both local and landscape scale.

### Common species of the metacommunity

The secondary forests of the Agua Salud landscape were characterized by a small group of widely distributed species. Only 7% of the total number of 526 plant species in our study occurred in at least 50% of our plots in any of the size classes. Even fewer species were frequently abundant. For example, only six species were widespread and abundant enough to make up more than 5 percent of all plants ≥ 1 cm diameter in more than 10% of the plots. In other words, most locally dominant species were not dominant across the metacommunity.

As we mentioned before, the changes in the frequencies of this small subset of species drove the observed patterns of deterministic compositional change in the metacommunity. The question of what characterizes these species is not easily answered [75]. Looking at figure 5, we can divide the very-frequent species roughly into three groups. We already discussed the group of initially very-frequent species that showed a strong decline in frequency later in succession. The group of species that were very frequent in both early and later successional stages encompasses a broader array of regeneration strategies and longevities, including short- and long-lived pioneers [76], [77]. While most species of this group did not or rarely regenerated in the oldest plots, *Miconia affinis* and *X. frutescens* were found as seedlings in 47% and 34% of the these plots, respectively. Species in a third group were infrequent across the earliest sites, but became very frequent later in succession. This group was even more diverse and included understory species such as *Swartzia simplex*, *Lacistema aggregatum*, canopy species such as *Pachira sessilis* and *Casearia arborea*, as well as pioneer species such as *Trichospermum galleottii* (Table S2).

These observations support previous studies that found that different life history strategies can lead to success during the first few decades of succession [20], [77]. What most of the very-frequent species in our metacommunity have in common, however, is that they produce copious amounts of small seeds that are dispersed by wind, birds, or bats. Together, these observations suggests a pivotal role for dispersal as determinant for which species become frequent across the metacommunity and abundant in the local communities [70], [78].

### The conservation value of secondary forests in a dynamic landscape

Understanding the conservation value of secondary forests involves two different questions: First, ***do successional changes in diversity and composition of the metacommunity indicate potential for forest recovery through natural succession in the long-term? ***In our site, the high plant species diversity in the metacommunity of early successional forests, including a wide array of life history strategies, suggests a resoundingly positive answer to this question.

It is, however, important to emphasize that this reflects the rich assemblage of seed sources in a land-use mosaic that is (i) adjacent to a large, contiguous tract of mature forest (PN Soberania) and (ii) includes mature forest patches, (iii) strips of old secondary forest and remnant trees along a dense network of streams and (iv) pastures with high densities of isolated trees and live fences. Our data shows that under such conditions a highly diverse assembly of plant species, including many mature forest species, can disperse to – and successfully establish in - secondary forests. These conditions represent the favorable end of a continuum of land-use scenarios in tropical landscapes, with large-scale and intensive land-use resulting in low tree cover, strong fragmentation and land degradation on the other end [4], [79].

The second question is ***to what extent can ephemeral secondary forests – fallows – in dynamic agricultural landscapes contribute to the conservation of a high diversity of tree species? ***This clearly depends on how old the secondary forests become and how many tree species will be able to reach reproductive size within that time frame. Unfortunately, data on size-related fecundity or a minimum reproductive size are only available for a handful of tropical tree species, even in the intensively-studied tropical forests of central Panama [59], [80], [81]. Using a reasonable size threshold of 30% to estimate size at reproductive maturity, our data suggest that the proportion of species that can sustain reproductive populations (effective diversity) in a dynamic patchwork of young secondary forests is, potentially, quite low. Also, the many occasional species that occur at very low frequencies in our metacommunity may be less likely to persist as viable meta-populations across the short-lived secondary forests in an agricultural matrix [82]. Removing these species from species counts indicates that young secondary forests (≤ 30 y) act as sink localities rather than as source localities for the metapopulations of most tree species [16], [83].

Moreover, our results illustrate that the species that will be able to maintain viable populations within the metacommunity of secondary forests will be a strongly biased sub-sample of the regional species pool. Most large canopy species, slow growing shade tolerant understory species, and species that depend on forest-dependent dispersers will not be part of that sub-group [4]. The impoverishment of the assemblage of large canopy species may have profound consequences for the long-term prospects of restoring forest structure and the provision of forest ecosystem services in human-modified landscapes [78], [84]. These findings add to the increasing body of literature on the erosion of diversity and the shift in the functional composition of forests in anthropogenic landscapes [70], [85]–[88].

This study indicates a high potential for the restoration of diverse forests through natural succession, when trees and fragments of older forests are maintained in the agricultural matrix and land is abandoned or set aside for a long period of time. Moreover, secondary forests may act as a buffer for remnant old-forest fragments, ameliorating edge effects, improving landscape connectivity, and extending source habitat for a subset of the regional species pool [5], [15], [89]. However, in dynamic landscapes across the Tropics, secondary forests are often ephemeral as they are cleared after short fallow periods, before most mature tropical forest species are able to grow to reproductive size [9], [90]. Our result suggests that in such cases, secondary forests play a limited role as a biodiversity reservoir for tree species, and contribute little in ameliorating the eminent extinction debt that human-modified landscapes are facing [25], [91]–[94].

## Supporting Information

Figure S1 **The Agua Salud Project area and distribution of study plots.** The Agua Salud landscape is a mosaic of secondary forests of different ages, pastures, and cultivated fields (See Fig. 1 in the main text). The black lines indicate the borders of the Agua Salud Project (ASP) area that is managed by the Smithsonian Tropical Research Institute. Colored rectangles indicate 0.1 ha Secondary Forest Dynamic (SFD) plots with stand basal areas of 0-11 (blue), 11-20 (green), 20-30 (orange), and >30 m2 ha-1 (red), respectively. The SFD sites were established on randomly selected slopes, with one plot on the upper section of the slope and one plot on the lower section of the slope. Not the whole ASP area was available for the SFD study: In 2008 a mixed-species reforestation experiment (light yellow shaded area) and a teak plantation (dark yellow shaded area) were established as well. The darker blue shaded area was and still is an active pasture and the lighter blue shaded area is currently young secondary forest but was initially planned to be managed as active pasture as well. The green area is within Soberania National Park that borders the Panama Canal.
(PDF)

Figure S2 **Layout of the Secondary Forest Dynamics plots of the Agua Salud Project.** In each site, two SFD plots were established, one up slope and one down slope, both perpendicular to the main slope direction (See Fig. S1). Total size of each plot is 20×50 m, divided in 5×5 m quadrants. Grey quadrants: All plants ≥ 1 cm DBH (Trees, shrubs, palms, lianas). White quadrants: all trees, shrubs and palms ≥ 5 cm DBH and lianas ≥ 1 cm DBH. Black 1×1 m quadrants: seedlings 20-80 cm height.
(PDF)

Figure S3 **Changes in site- and landscape level community structure with age since abandonment.** Changes in community structure with time since abandonment and with light were assessed for different plant groups using several indices of species composition and diversity. Seedlings included palms and lianas, while saplings and trees included palms but not lianas. ^0^D  =  number of species per unit sample area; ^1^D  =  the exponential of Shannon entropy; ^2^D  =  the inverse Simpson concentration. Composition  =  the SFD site score on the first axis of an NMDS ordination of the plant communities, computed with the abundance-based Jaccard dissimilarity index (white dots) and the Jaccard index based on presence/absence data (orange dots). Sample area per plot and per SBA class is indicated above the graphs. Equation type and regression statistics for all relationships are given in Table 2 in the main text.
(PDF)

Figure S4 **Relationships between dissimilarities in species composition and spatial distance.** Mantel correlation as function of distance between SFD plots for (A) the 2-7 y age class and (B) the 0-10 m^2^ha^−1^ stand basal area class. Red dots and lines: seedlings 20-80 cm height; Blue dots and lines: saplings 1-5 cm DBH; Orange dots and lines: trees > 5 cm DBH; Green dots and lines: lianas > 1 cm diameter. Filled dots indicate significance (P < 0.05).
(PDF)

Figure S5 **Changes in rarefied species richness during succession**. Rarefied species richness as a function of successional stand development, for 4 different plant groups. a-d) Plot-level species richness was rarefied to the average number of species per 12 individuals, which was the lowest number of individuals among all plant group × plot combinations. Error bars were too small to show. e) On landscape scale, species richness was rarefied to the average number of species per 1400 individuals, which was the lowest number of stems among all plant group × SBA class combination. Error bars indicate the 95% confidence interval. Colors indicate plant groups as in S5a-d.
(PDF)

Table S1 **List of species with growth form and abundances in two size classes**.
(TXT)

Table S2 **Frequency of occurrence in stand basal area classes.** List of species that occur, with individuals ≥ 1 cm diameter, in (i) at least 50% of 98 SFD plots and/or in (ii) at least one of the stand basal area (SBA) classes with at least 33% of SFD plots with ≥ 5% of individuals. Species are ordered on their frequency of occurrence with ≥ 5% of all individuals in SBA-1, SBA-2 and SBA-3, respectively. CTFS  =  permanent species code used in the databases of the Centre of Tropical Forest Science; GF  =  growth form: Tc  =  canopy species; Tm  =  mid story tree species; Tu  =  understory tree species; S  =  shrub species; L  =  liana species; P  =  palm species.
(PDF)
