# Supplementary material for: Succession of Ephemeral Secondary Forests and Their Limited Role for the Conservation of Floristic Diversity in a Human-Modified Tropical Landscape
Source: PLoS One. 2013 Dec 11;8(12):e82433. doi: 10.1371/journal.pone.0082433 (PMC3859589; doi:10.1371/journal.pone.0082433)
Supplement: Figure S1 — The Agua Salud Project area and distribution of study plots. The Agua Salud landscape is a mosaic of secondary forests of different ages, pastures, and cultivated fields (See Fig. 1 in the main text). The black lines indicate the borders of the Agua Salud Project (ASP) area that is managed by the Smithsonian Tropical Research Institute. Colored rectangles indicate 0.1 ha Secondary Forest Dynamic (SFD) plots with stand basal areas of 0-11 (blue), 11-20 (green), 20-30 (orange), and >30 m2 ha-1 (red), respectively. The SFD sites were established on randomly selected slopes, with one plot on the upper section of the slope and one plot on the lower section of the slope. Not the whole ASP area was available for the SFD study: In 2008 a mixed-species reforestation experiment (light yellow shaded area) and a teak plantation (dark yellow shaded area) were established as well. The darker blue shaded area was and still is an active pasture and the lighter blue shaded area is currently young secondary forest but was initially planned to be managed as active pasture as well. The green area is within Soberania National Park that borders the Panama Canal. (PDF) [file pone.0082433.s001.pdf]

**Supporting Information**

## Figure S1 | The Agua Salud Project area and distribution of study plots

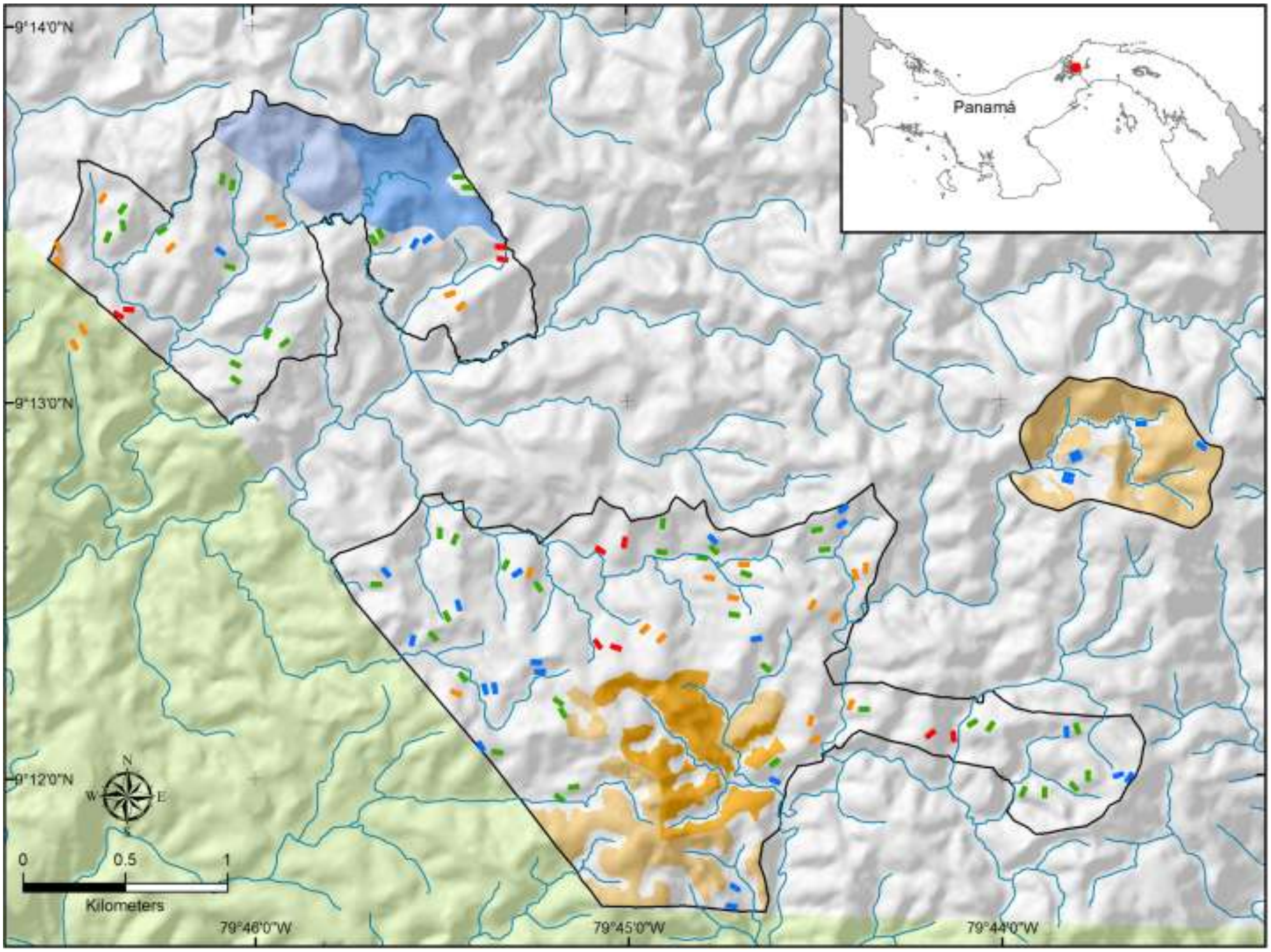


The Agua Salud landscape is a mosaic of secondary forests of different ages, pastures, and cultivated fields (See Fig. 1 in the main text). The black lines indicate the borders of the Agua Salud Project (ASP) area that is managed by the Smithsonian Tropical Research Institute. Colored rectangles indicate 0.1 ha Secondary Forest Dynamic (SFD) plots with stand basal areas of 0-11 (blue), 11-20 (green), 20-30 (orange), and >30 m2 ha-1 (red), respectively. The age of the latter sites was estimated to range between 50 and 80 years, but no information was available to confirm this estimate. Furthermore, no reliable information was available on forest use since abandonment. We therefore decided to exclude these sites from analyses. The SFD sites were established on randomly selected slopes, with one plot on the upper section of the slope and one plot on the lower section of the slope. Not the whole ASP area was available for the SFD study: In 2008 a mixed-species reforestation experiment (light yellow shaded area) and a teak plantation (dark yellow shaded area) were established as well. The darker blue shaded area was and still is an active pasture and the lighter blue shaded area is currently young secondary forest but was initially planned to be managed as active pasture as well. The green area is within Soberania National Park that borders the Panama Canal.
