# Supplementary material for: Succession of Ephemeral Secondary Forests and Their Limited Role for the Conservation of Floristic Diversity in a Human-Modified Tropical Landscape
Source: PLoS One. 2013 Dec 11;8(12):e82433. doi: 10.1371/journal.pone.0082433 (PMC3859589; doi:10.1371/journal.pone.0082433)

Supporting Information

Figure S2 | Layout of the Secondary Forest Dynamics plots of the Agua Salud Project

In each site, two SFD plots were established, one up slope and one down slope, both perpendicular to the main slope direction (See Fig. S1). Total size of each plot is 20 × 50 m, divided in 5 × 5 m quadrants. Grey quadrants: All plants ≥ 1 cm DBH (Trees, shrubs, palms, lianas). White quadrants: all trees, shrubs and palms ≥ 5 cm DBH and lianas ≥ 1 cm DBH. Black 1×1 m quadrants: seedlings 20-80 cm height.

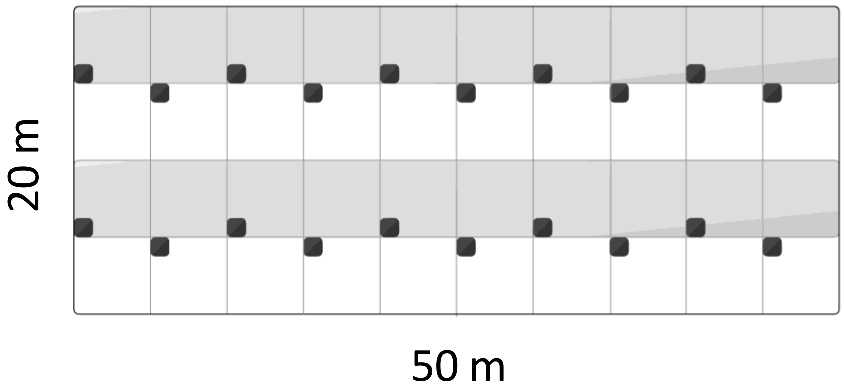

Supplement: Figure S2 — Layout of the Secondary Forest Dynamics plots of the Agua Salud Project. In each site, two SFD plots were established, one up slope and one down slope, both perpendicular to the main slope direction (See Fig. S1). Total size of each plot is 20×50 m, divided in 5×5 m quadrants. Grey quadrants: All plants ≥ 1 cm DBH (Trees, shrubs, palms, lianas). White quadrants: all trees, shrubs and palms ≥ 5 cm DBH and lianas ≥ 1 cm DBH. Black 1×1 m quadrants: seedlings 20-80 cm height. (PDF) [file pone.0082433.s002.pdf]
