# Supplementary material for: Succession of Ephemeral Secondary Forests and Their Limited Role for the Conservation of Floristic Diversity in a Human-Modified Tropical Landscape
Source: PLoS One. 2013 Dec 11;8(12):e82433. doi: 10.1371/journal.pone.0082433 (PMC3859589; doi:10.1371/journal.pone.0082433)
Supplement: Figure S3 — Changes in site- and landscape level community structure with age since abandonment. Changes in community structure with time since abandonment and with light were assessed for different plant groups using several indices of species composition and diversity. Seedlings included palms and lianas, while saplings and trees included palms but not lianas. 0D = number of species per unit sample area; 1D = the exponential of Shannon entropy; 2D = the inverse Simpson concentration. Composition = the SFD site score on the first axis of an NMDS ordination of the plant communities, computed with the abundance-based Jaccard dissimilarity index (white dots) and the Jaccard index based on presence/absence data (orange dots). Sample area per plot and per SBA class is indicated above the graphs. Equation type and regression statistics for all relationships are given in Table 2 in the main text. (PDF) [file pone.0082433.s003.pdf]

Figure S3 | Changes in site- and landscape level community structure with age since abandonment

---

Changes in community structure with time since abandonment and with light were assessed for different plant groups using several indices of species composition and diversity. Seedlings included palms and lianas, while saplings and trees included palms but not lianas. ^{0}D = number of species per unit sample area; ^{1}D = the exponential of Shannon entropy; ^{2}D = the inverse Simpson concentration. See the method section of the main text for more details. Composition = the SFD site score on the first axis of an NMDS ordination of the plant communities, computed with the abundance-based Jaccard dissimilarity index (white dots) and the Jaccard index based on presence / absence data (orange dots). Sample area per plot and per SBA class is indicated above the graphs. Equation type and regression statistics for all relationships are given in Table 2 in the main text.

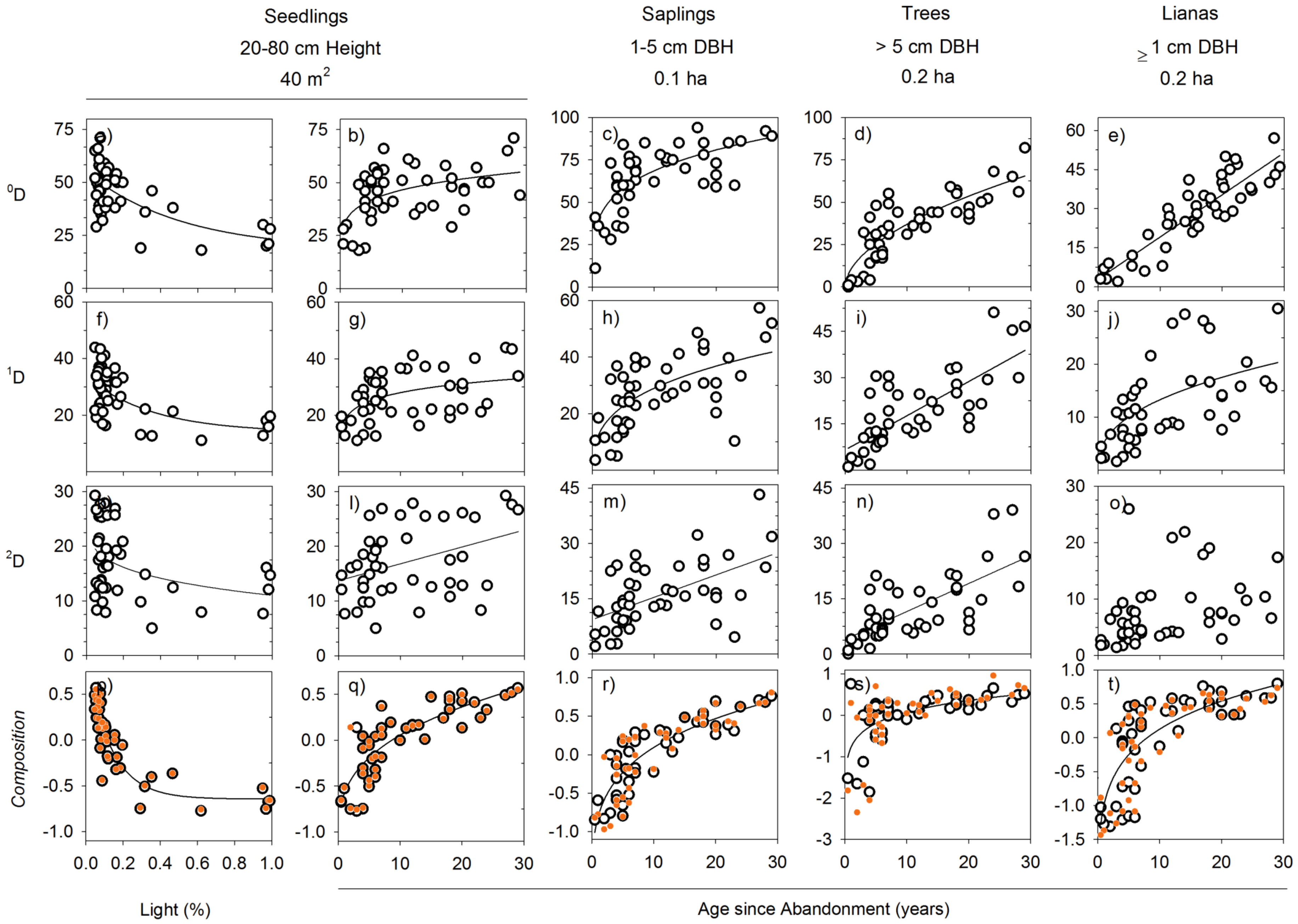
Seedlings
20-80 cm Height
40 m2
Saplings
1-5 cm DBH
0.1 ha
Trees
> 5 cm DBH
0.2 ha
Lianas
≥ 1 cm DBH
0.2 ha
0D
1D
2D
Composition
Light (%)
Age since Abandonment (years)
