# Supplementary material for: Succession of Ephemeral Secondary Forests and Their Limited Role for the Conservation of Floristic Diversity in a Human-Modified Tropical Landscape
Source: PLoS One. 2013 Dec 11;8(12):e82433. doi: 10.1371/journal.pone.0082433 (PMC3859589; doi:10.1371/journal.pone.0082433)
Supplement: Figure S4 — Relationships between dissimilarities in species composition and spatial distance. Mantel correlation as function of distance between SFD plots for (A) the 2-7 y age class and (B) the 0-10 m2ha−1 stand basal area class. Red dots and lines: seedlings 20-80 cm height; Blue dots and lines: saplings 1-5 cm DBH; Orange dots and lines: trees > 5 cm DBH; Green dots and lines: lianas > 1 cm diameter. Filled dots indicate significance (P < 0.05). (PDF) [file pone.0082433.s004.pdf]

Supporting Information

Figure S4 | Relationships between dissimilarities in species composition and spatial distance

Mantel correlation between dissimilarities in the species composition of - and the spatial distance between the SFD plots was calculated separately for each of four plant groups × three age classes and for each of four plant groups × three stand basal area classes. *Plant groups* were: seedlings 20-80 cm height (red dots and lines), saplings 1-5 cm DBH (blue dots and lines), trees > 5 cm DBH (orange dots and lines) and lianas > 1 cm diameter (green dots and lines). *Age classes* were: 2-7y, 8-17y and 18-34y. *Stand basal area classes* were: 0-10m^2ha^{-1}, 10.1-20m^2ha^{-1} and 20.1-30m^2ha^{-1}.

Mantel statistics [1] were calculated using the function 'mantel' and 'mantel.correlog' of the R package 'vegan' based on Pearson's product-moment correlation [2]. The species dissimilarity matrices were generated from the Chao-Jaccard abundance index and with the Chao Jaccard abundance estimator, using the 'vegdist' function of the vegan package [2]. Both indices yielded similar results, with slightly lower mantel correlations for the latter. Here we present the data based on the former. The significance of the mantel statistic was evaluated by permuting rows and columns of the species dissimilarity matrix 999 times [2].

For all plant groups, the mantel correlation was only significant ($P < 0.05$) in the earliest successional stage. In the 2-7 y age class, correlations were 0.17, 0.27, 0.14 and 0.33 for seedlings, saplings, trees and lianas, respectively. In the 0-10 m^2ha^{-1} stand basal area class, values were 0.13, 0.26, 0.13 and 0.30, respectively. In **figure S4**, the Mantel correlation is plotted as function of distance between SFD plots for (**A**) the 2-7 y age class and (**B**) the 0-10 m^2ha^{-1} stand basal area class. Filled dots indicate significance ($P < 0.05$).

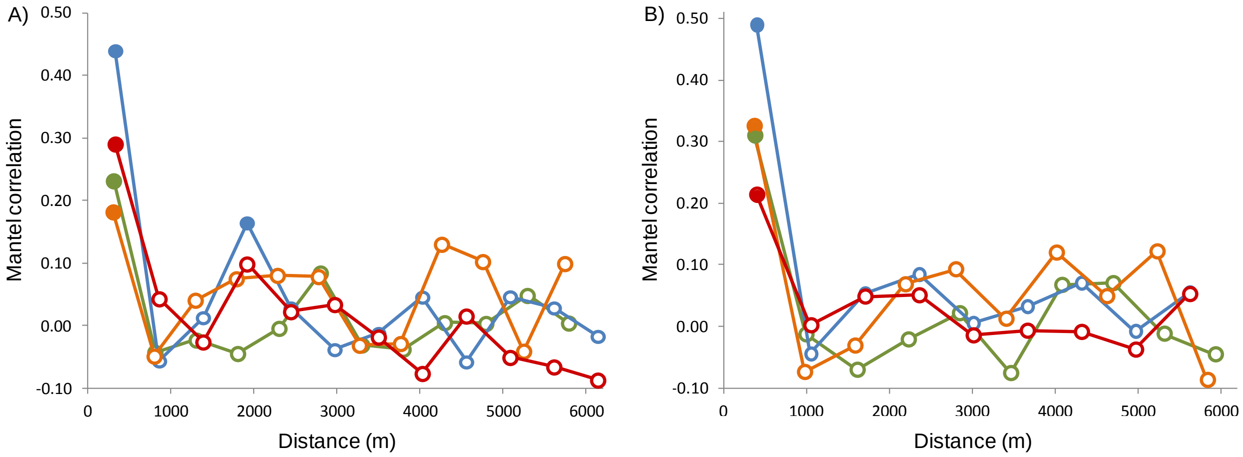

References

1. Legendre, P. and Legendre, L. (2012) Numerical Ecology. 3rd English Edition. Elsevier.
2. Oksanen J, Blanchet FG, Kindt R, Legendre P, Minchin PR, et al. (2003) vegan: Community Ecology Package. Available: http://cran.r-project.org/package=vegan.
