# Supplementary material for: Succession of Ephemeral Secondary Forests and Their Limited Role for the Conservation of Floristic Diversity in a Human-Modified Tropical Landscape
Source: PLoS One. 2013 Dec 11;8(12):e82433. doi: 10.1371/journal.pone.0082433 (PMC3859589; doi:10.1371/journal.pone.0082433)

Figure S5 | Changes in rarefied species richness during succession

Rarefied species richness as a function of successional stand development, for 4 different plant groups. **a-d**) Plot-level species richness was rarefied to the average number of species per 12 individuals, which was the lowest number of individuals among all plant group × plot combinations. Error bars were too small to show. **e**) On landscape scale, species richness was rarefied to the average number of species per 1400 individuals, which was the lowest number of stems among all plant group × SBA class combination. Error bars indicate the 95% confidence interval. Colors indicate plant groups as in Fig. S5a-d. Rarefaction was performed using the function 'rarefy' of the R package 'vegan'.

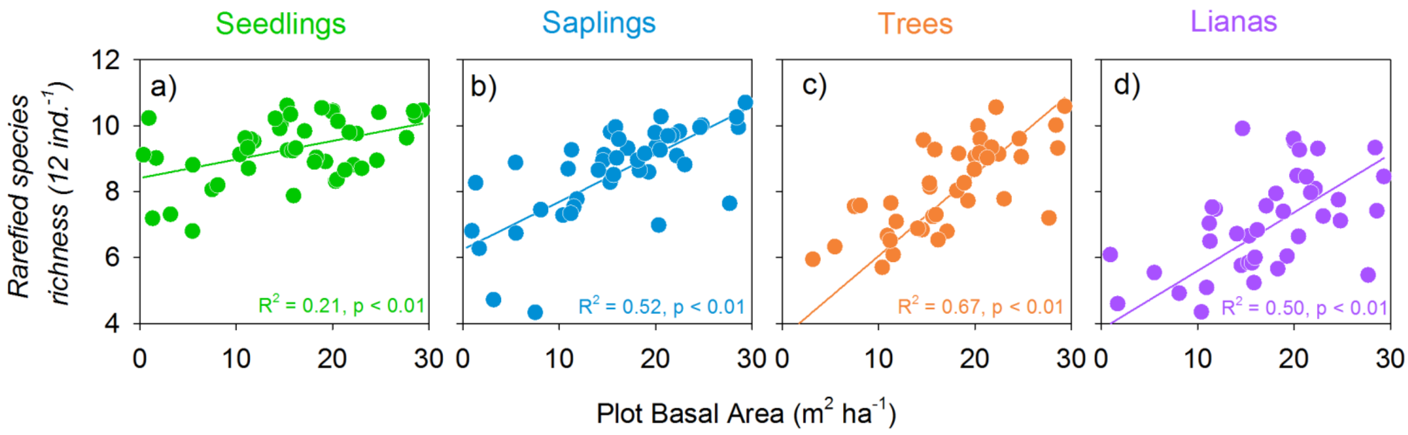

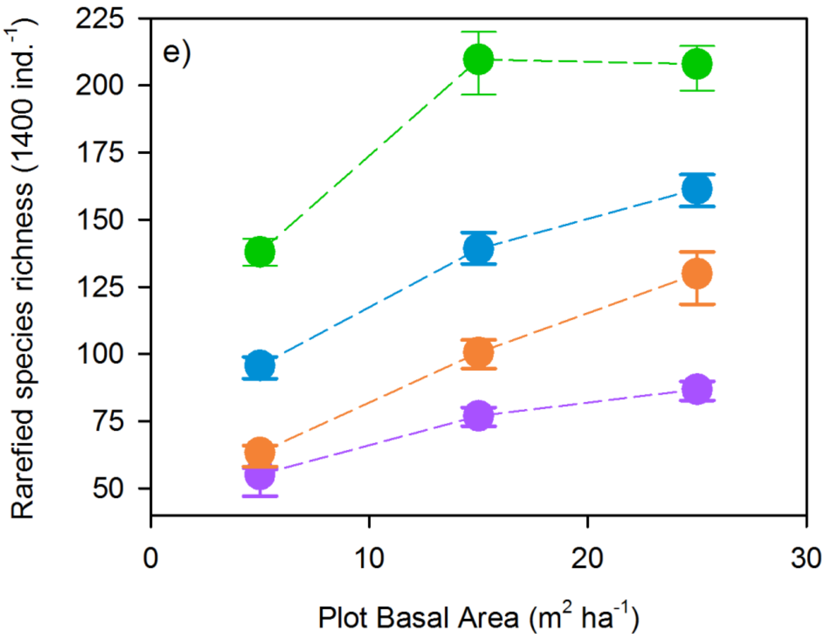

Supplement: Figure S5 — Changes in rarefied species richness during succession. Rarefied species richness as a function of successional stand development, for 4 different plant groups. a-d) Plot-level species richness was rarefied to the average number of species per 12 individuals, which was the lowest number of individuals among all plant group × plot combinations. Error bars were too small to show. e) On landscape scale, species richness was rarefied to the average number of species per 1400 individuals, which was the lowest number of stems among all plant group × SBA class combination. Error bars indicate the 95% confidence interval. Colors indicate plant groups as in S5a-d. (PDF) [file pone.0082433.s005.pdf]
