# Supplementary material for: Succession of Ephemeral Secondary Forests and Their Limited Role for the Conservation of Floristic Diversity in a Human-Modified Tropical Landscape
Source: PLoS One. 2013 Dec 11;8(12):e82433. doi: 10.1371/journal.pone.0082433 (PMC3859589; doi:10.1371/journal.pone.0082433)
Supplement: Table S2 — Frequency of occurrence in stand basal area classes. List of species that occur, with individuals ≥ 1 cm diameter, in (i) at least 50% of 98 SFD plots and/or in (ii) at least one of the stand basal area (SBA) classes with at least 33% of SFD plots with ≥ 5% of individuals. Species are ordered on their frequency of occurrence with ≥ 5% of all individuals in SBA-1, SBA-2 and SBA-3, respectively. CTFS = permanent species code used in the databases of the Centre of Tropical Forest Science; GF = growth form: Tc = canopy species; Tm = mid story tree species; Tu = understory tree species; S = shrub species; L = liana species; P = palm species. (PDF) [file pone.0082433.s007.pdf]

Supporting Information

Table S2 | Frequency of occurrence in stand basal area classes

List of species that occur, with individuals ≥ 1 cm diameter, in at least 50% of 98 SFD plots and/or in at least one of the stand basal area (SBA) classes with 33% of SFD plots with ≥ 5% of individuals. Species are ordered on their frequency of occurrence with ≥ 5% of all individuals in SBA-1, SBA-2 and SBA-3, respectively. CTFS = permanent species code used in the databases of the Centre of Tropical Forest Science; GF = growth form: Tc = canopy species; Tm = mid story tree species; Tu = understory tree species; S = shrub species; L = liana species; P = palm species.

						Frequency of occurrence (% of SFD plots)					
						with >= plants			with >= 5% of plants		
**Nr.**	**CTFS**	**Family**	**Genus**	**Species**	**GF**	**SBA-1**	**SBA-2**	**SBA-3**	**SBA-1**	**SBA-2**	**SBA-3**
1	CONOXA	Melastomataceae	Conostegia	xalapensis	Tu	93	92	47	68	21	13
2	VISMBA	Clusiaceae	Vismia	baccifera	Tu	93	97	69	54	34	6
3	DAVINI	Dilleniaceae	Davilla	nitida	L	75	100	100	36	71	66
4	VERNPA	Asteraceae	Vernonanthura	patens	Tu	93	42	3	36	3	
5	VISMMA	Clusiaceae	Vismia	macrophylla	Tm	93	92	97	32	21	3
6	BYRSCR	Malpighiaceae	Byrsonima	crassifolia	Tc	96	92	66	25	11	6
7	SENNHA	Fabaceae:Caesal.	Senna	hayesiana	S	71	61	25	21	3	
8	MICOAR	Melastomataceae	Miconia	argentea	Tm	89	100	97	18	50	19
9	XYL1FR	Annonaceae	Xylopia	frutescens	Tm	82	100	94	14	26	31
10	THEVAH	Apocynaceae	Thevetia	ahouai	Tu	32	66	75	14	18	16
11	PIPEAD	Piperaceae	Piper	aduncum	S	68	29	3	14	3	
12	BANAGU	Salicaceae	Banara	guianensis	Tu	64	68	34	11	5	
13	MICOBO	Melastomataceae	Miconia	minutiflora	Tm	57	61	38	11	5	
14	CUPACI	Sapindaceae	Cupania	cinerea	Tm	46	66	53	7	3	3
15	ADENFL	Lythraceae	Adenaria	floribunda	S	50	16	9	7		
16	CONOSP	Melastomataceae	Conostegia	speciosa	S	61	29	9	7		
17	HET1VI	Asteraceae	Heterocondylus	vitalbae	L	46	53	13	7		
18	LANTCA	Verbenaceae	Lantana	camara	S	57	5		7		
19	PALIGU	Rubiaceae	Palicourea	guianensis	S	64	89	69	4	5	3
20	LEPICA	Asteraceae	Lepidaploa	canescens	L	82	34		4		
21	SIPAPA	Monimiaceae	Siparuna	pauciflora	Tu	11	39	50	4		
22	MICOAF	Melastomataceae	Miconia	affinis	Tu	54	82	91		18	9
23	CONNPA	Connaraceae	Connarus	panamensis	L	36	87	94		13	16

						Frequency of occurrence (% of SFD plots)					
						with >= plants			with >= 5% of plants		
Nr.	CTFS	Family	Genus	Species	GF	SBA-1	SBA-2	SBA-3	SBA-1	SBA-2	SBA-3
24	LACIAG	Lacistemataceae	Lacistema	aggregatum	Tu	29	92	97		11	28
25	RYANSP	Salicaceae	Ryania	speciosa	Tu	18	58	78		5	13
26	CASESY	Salicaceae	Casearia	sylvestris	Tm	64	84	97		5	3
27	COCHVI	Cochlospermaceae	Cochlospermum	vitifolium	Tu	29	55	63		5	3
28	PHRYCO	Bignoniaceae	Phryganocydia	corymbosa	L	7	50	72		5	3
29	VISMBI	Clusiaceae	Vismia	billbergiana	Tu	32	53	41		5	3
30	TRI4GA	Malvaceae	Trichospermum	galeottii	Tm	43	63	75		5	
31	CUPASC	Sapindaceae	Cupania	scrobiculata	Tm	25	53	63		3	13
32	GOUAPO	Rhamnaceae	Gouania	polygama	L	25	55	44		3	3
33	INGACO	Fabaceae:Mimos.	Inga	cocleensis	Tm	46	84	88		3	3
34	MAR2PA	Convolvulaceae	Maripa	panamensis	L		42	75		3	3
35	PSYCG3	Rubiaceae	Psychotria	grandis	Tu	18	32	50		3	3
36	QUASAM	Simaroubaceae	Quassia	amara	Tu	11	50	63		3	3
37	TALINE	Sapindaceae	Talisia	nervosa	Tu	4	53	72		3	3
38	APEITI	Malvaceae	Apeiba	tibourbou	Tm	21	63	81		3	
39	INGATH	Fabaceae:Mimos.	Inga	thibaudiana	Tm	29	68	69		3	
40	MICOPO	Melastomataceae	Miconia	poeppigii	Tc	4	16	53		3	
41	AMAICO	Rubiaceae	Amaioua	corymbosa	Tu	4	37	59			6
42	DOLIMU	Dilleniaceae	Doliocarpus	multiflorus	L	29	45	66			6
43	ANNOSP	Annonaceae	Annona	spraguei	Tm	46	63	78			3
44	BACTC1	Arecaceae	Bactris	coloniata	P		26	63			3
45	SABIPA	Rubiaceae	Sabicea	panamensis	L	11	42	63			3
46	ALCHCO	Euphorbiaceae	Alchornea	costaricensis	Tc	39	58	47			
47	BROSGU	Moraceae	Brosimum	guianense	Tc		37	78			
48	BYRSSP	Malpighiaceae	Byrsonima	spicata	Tc	7	37	59			
49	CASEAR	Salicaceae	Casearia	arborea	Tc	11	34	63			
50	CASECO	Salicaceae	Casearia	commersoniana	Tu	32	74	84			
51	CESTME	Solanaceae	Cestrum	megalophyllum	S	4	24	53			
52	CNESRU	Connaraceae	Cnestidium	rufescens	L	46	79	72			
53	CORDBI	Boraginaceae	Cordia	bicolor	Tc	32	55	72			
54	CUPARU	Sapindaceae	Cupania	rufescens	Tc	32	58	63			
55	DIO1ME	Dioscoreaceae	Dioscorea	mexicana	L	11	26	53			
56	DOLIDE	Dilleniaceae	Doliocarpus	dentatus	L	21	66	59			

						Frequency of occurrence (% of SFD plots)					
						with >= plants			with >= 5% of plants		
**Nr.**	**CTFS**	**Family**	**Genus**	**Species**	**GF**	**SBA-1**	**SBA-2**	**SBA-3**	**SBA-1**	**SBA-2**	**SBA-3**
57	DOLIMA	Dilleniaceae	Doliocarpus	major	L	18	58	56			
58	FORSVI	Apocynaceae	Forsteronia	viridescens	L		32	66			
59	INGASE	Fabaceae:Mimos.	Inga	sertulifera	Tu	4	34	50			
60	LAXOTE	Apocynaceae	Laxoplumeria	tessmannii	Tm	11	50	66			
61	MACHFL	Fabaceae	Machaerium	floribundum	L	7	24	59			
62	MARGNO	Phyllanthaceae	Margaritaria	nobilis	Tm	11	34	53			
63	MYR2FL	Myrtaceae	Myrciaria	floribunda	Tu	21	42	63			
64	OCOTDE	Lauraceae	Ocotea	dendrodaphne	Tm	7	32	56			
65	POCHSE	Bombacaceae	Pachira	sessilis	Tc	25	50	84			
66	PAULRU	Sapindaceae	Paullinia	rugosa	L	7	42	78			
67	PENTMA	Rubiaceae	Pentagonia	macrophylla	Tu	14	39	50			
68	PETRAS	Verbenaceae	Petrea	volubilis	L	11	26	63			
69	PROTPA	Burseraceae	Protium	panamense	Tm	4	29	50			
70	ROURGL	Connaraceae	Rourea	glabra	L		50	69			
71	SCH2MO	Araliaceae	Schefflera	morototoni	Tc	39	74	66			
72	SWARS1	Fabaceae:Papil.	Swartzia	simplex	Tu	7	50	78			
73	TERMAM	Combretaceae	Terminalia	amazonia	Tc	86	87	75			
74	TURPOC	Staphyleaceae	Turpinia	occidentalis	Tc	7	34	56			
75	ZANTPR	Rutaceae	Zanthoxylum	acuminatum	Tm	4	39	66			
